# Litrre’s hernia: partially reduced inguinal hernia in a 3-year-old boy

**DOI:** 10.1093/jscr/rjab421

**Published:** 2021-09-30

**Authors:** Bardisan Gawrieh, Waseem Shater, Mohammad Ali Deeb, Alaa Ghuzlan, Hanna Kassab, Nabila Salhab

**Affiliations:** Pediatric Surgery Department, Tishreen University Hospital, Lattakia, Syria; Cancer Research Center, Tishreen University, Lattakia, Syria; Pediatric Surgery Department, Tishreen University Hospital, Lattakia, Syria; Pediatric Surgery Department, Tishreen University Hospital, Lattakia, Syria; Pediatric Surgery Department, Tishreen University Hospital, Lattakia, Syria; Pediatric Surgery Department, Tishreen University Hospital, Lattakia, Syria; Pediatric Surgery Department, Tishreen University Hospital, Lattakia, Syria

## Abstract

This report examines the case of a 3-year-old child presenting with a 1-month history of swelling in the right groin. The boy had no associated nausea or vomiting, was afebrile and had had normal bowel movements. Attempts to reduce the swelling were only partially successful. Ultrasonography indicated the presence of turbid hydrocele and a hernia sac containing an intestinal loop. Accordingly, the patient underwent an urgent herniotomy. Exposing the hernia sac revealed 5 cm Meckel’s diverticulum, and the base of the diverticulum was resected from the inside of the hernia sac. The boy was discharged 4 days after the operation in good clinical condition. The presented case highlights the need to consider Littre’s hernia when dealing with partially reduced inguinal hernias in children with no general signs or evidence of intestinal obstruction.

## INTRODUCTION

Meckel’s diverticulum (MD) is the most prevalent congenital anomaly of the gastrointestinal tract, occurring in 2% of the population with a 2: 1 male predominance. Approximately 2% of patients develop complications over the course of their lives, typically before the age of 2 [1]. The most frequent clinical presentation in pediatric population is hemorrhage [2], and Littre’s hernia is a rare complication (4–10%) [1]. It was originally defined by Reinke in 1841 as the presence of a MD in any hernia sac [3], with the common site (50% of cases) being the inguinal canal, usually on the right [1].

## CASE REPORT

**Figure 1:**
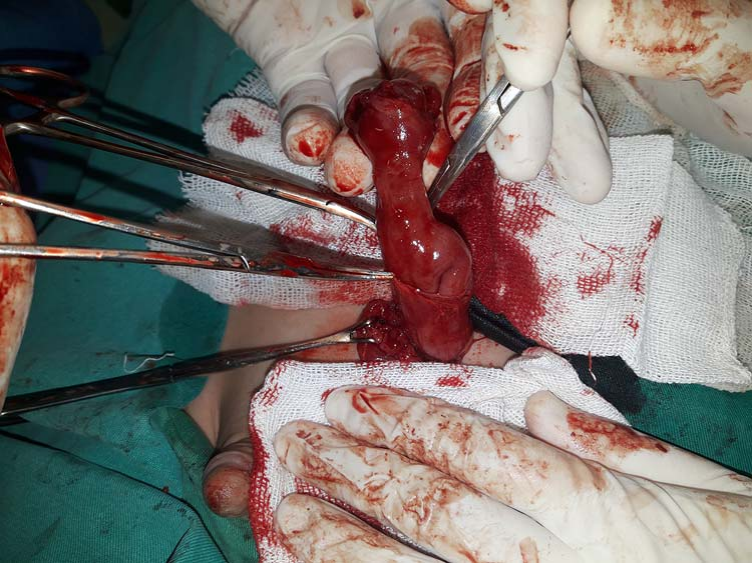
Exposing the hernia sac revealed a 5 cm MD.

**Figure 2:**
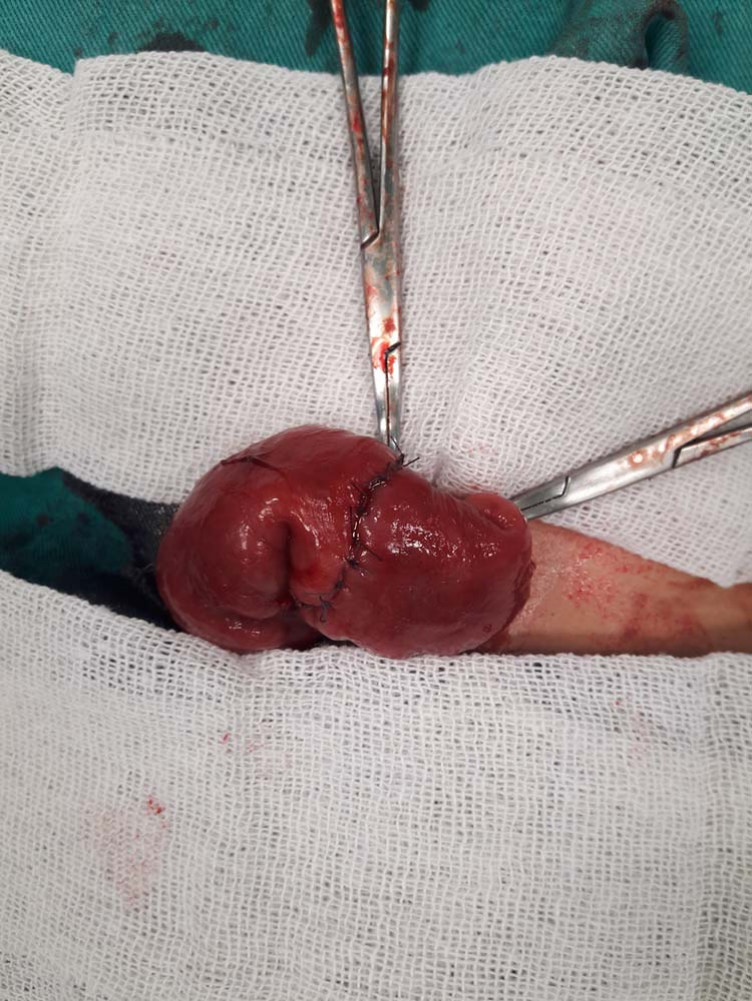
Ileoileal primary anastomosis

**Figure 3:**
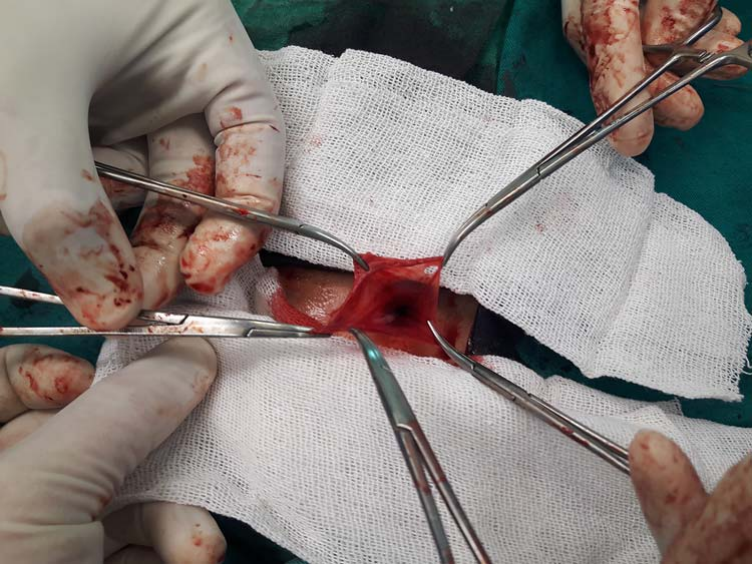
Hernia sac before ligation.

**Figure 4:**
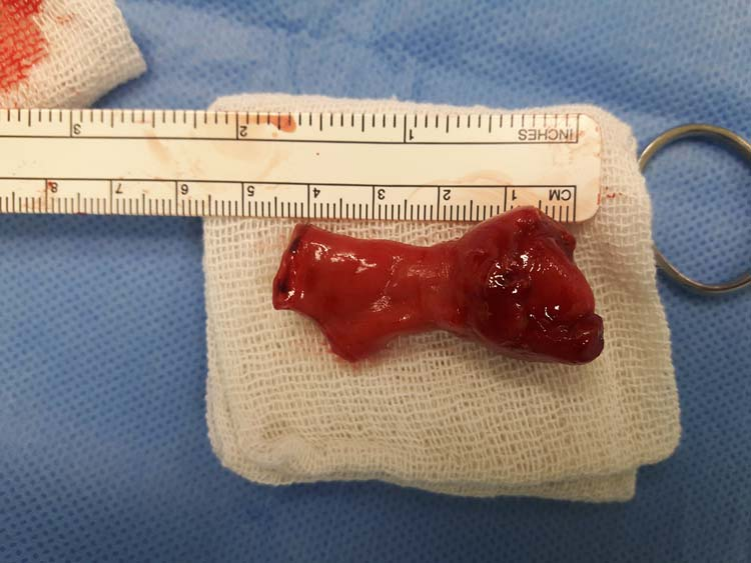
MD after resection.

**Figure 5:**
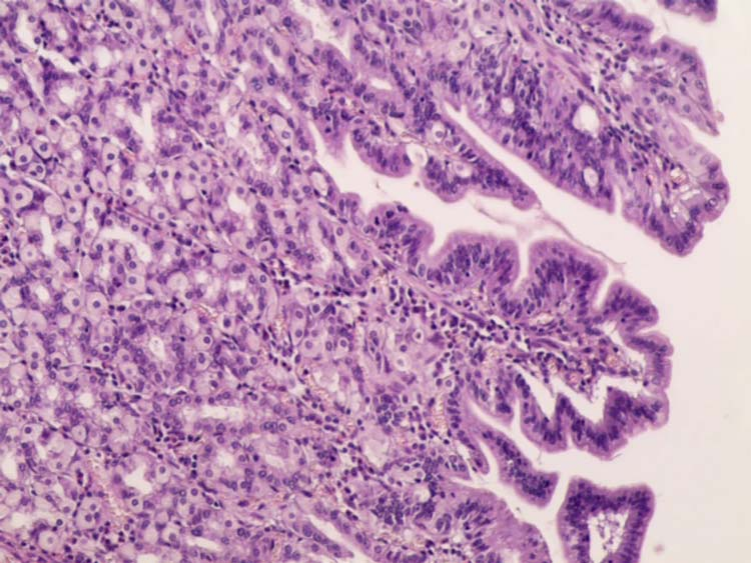
Microscopic image.

**Figure 6:**
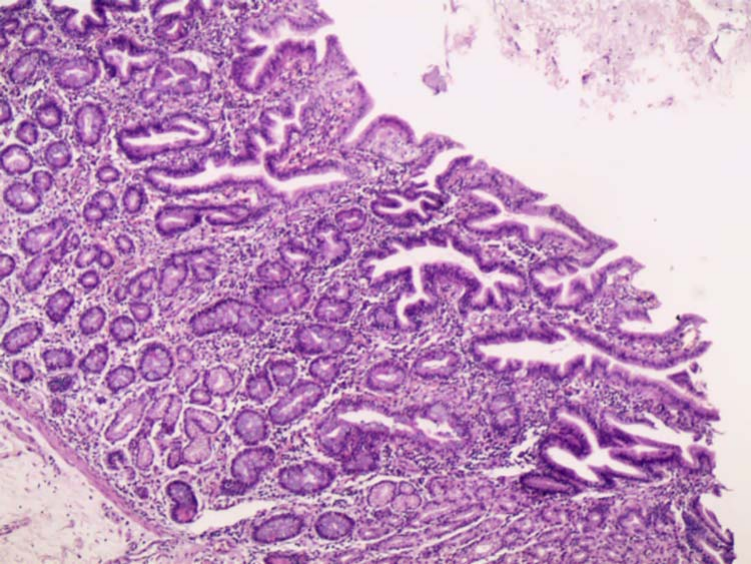
Microscopic image.

**Figure 7:**
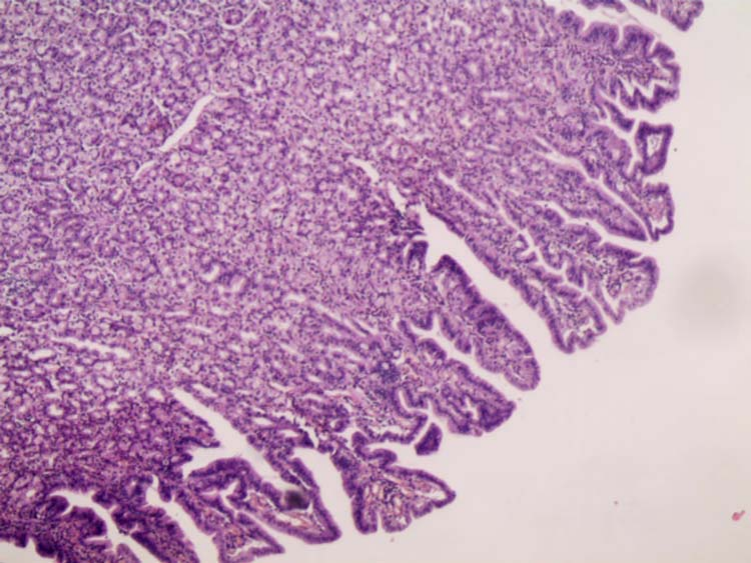
Microscopic image.

A 3-year-old Syrian boy with a history of right herniorrhaphy at 1 year of age was admitted to the department with complaints of left-sided groin swelling. Physical examination revealed a firm swelling in the left groin that was partially reduced with no associated skin changes. The boy had no associated nausea or vomiting, was afebrile and had had normal bowel movements. Inflammatory markers were normal. Ultrasonography indicated the presence of turbid hydrocele and a hernia sac containing an intestinal loop with normal blood supply. Accordingly, the patient underwent an urgent herniotomy the next following day. The mass was approached with a groin area incision and exposing the hernia sac revealed a 5 cm MD anchored to it (Fig. 1). Wedge-shaped resection of the base of the diverticulum was performed from the inside of the hernia sac. High ligation of the hernia sac was completed after the intestinal contents were addressed to the intraperitoneal cavity and performing the anastomosis (Figs 2–4). Early enteral nutrition was started 20 hours postoperatively with gradual increase until goal feedings were reached. The immediate postoperative course was uneventful, and the patient did not present any symptoms during the postoperative follow-up. Microscopic examination showed that the bulge was MD with ectopic gastric mucosa, and no malignancy was observed after a thorough examination (Figs 5–7).

## DISCUSSION

Indirect inguinal hernias are the most commonly incarcerated hernias in children. An incarcerated hernia presents as an irreducible non-fluctuant bulge that is tender and may be erythematous. Conditions that may be confused for an incarcerated hernia include retractile testis, lymphadenopathy and hydrocele. Reduction with or without sedation is possible in one-quarter to one-third of cases and normally leads to delayed surgery [4, 5]. During incarceration of Littre hernia, all symptoms of strangulated hernia in infancy may be present, and there is no specific sign that advocates the presence of MD in a hernia sac [5]. Our patient presented with partially reduced inguinal hernia but showed no general signs or evidence of intestinal obstruction. Despite all surgical improvements, the most significant challenge is still the preoperative diagnosis of Littre hernia because it can mimic an incarcerated and strangulated inguinal hernia [5, 6]. Even though neither abdominal ultrasonography nor computed tomography reaches a definite diagnosis, both can be used to differentiate between cases requiring surgical management and those that can be treated conservatively when dealing with irreducible inguinal hernia [7].

In our patient’s case, we had an ultrasound, which indicated a healthy intestinal loop herniated through the sac and the presence of turbid hydrocele. However, Littre’s hernia was not initially suspected.

The most usual treatment for Littre’s hernia is wedge-shaped resection of the base of the diverticulum from the inside of the hernial sac. Ileal resection and anastomosis should only be used in cases with severe damage to the loop or when fibrosis or ulceration of the base of the diverticulum is present, so as to prevent other complications due to the presence of heterotopic tissue. However, there is no evidence that segmental resection has any disadvantages over wedge resection or simple diverticulotomy in terms of complications or follow-up results [3, 5, 6, 8]. Both Laparoscopic and open repair techniques have been compared in the management of incarcerated inguinal hernias and are reported to have similar outcomes [4]. In the reported case, the open repair technique was carried out through a transverse inguinal incision and wedge-shaped resection of the base of the diverticulum performed from the inside of the hernia sac. Early enteral feeding in pediatric intestinal anastomosis can be safely started without waiting for traditional markers of return of bowel activity [9]. In our case, liquid feeds were initially started 20 hours postoperatively and were increased at 4-hour increments until goal feedings were reached. The patient did not present any symptoms during postoperative follow-up.

In conclusion, very few cases of children with Littre’s hernia have been reported in the literature and none could be preoperatively defined. Because its progress is more gradual compared to other hernias, Littre’s hernia should be taken into consideration if these three characteristics associate in children: (i) hernia contents are partly reduced, (ii) no general signs or evidence of intestinal obstruction and (iii) ultrasound indicates a healthy intestinal loop.

## CONFLICT OF INTEREST STATEMENT

The authors did not receive any funding to conduct this study.

## FUNDING

The authors declare that they have no known competing financial interests or personal relationships that could have influenced the work reported in this paper.
